# Hemodialysis Effect on the Composition of the Eye Fluid of Cataract Patients

**DOI:** 10.3390/jcm10235485

**Published:** 2021-11-23

**Authors:** Joanna Dolar-Szczasny, Jolanta Flieger, Beata Kowalska, Dariusz Majerek, Małgorzata Tatarczak-Michalewska, Izabela Zakrocka, Wojciech Załuska, Robert Rejdak

**Affiliations:** 1 Chair and Department of General and Pediatric Ophthalmology, Medical University of Lublin, Chmielna 1, 20-079 Lublin, Poland; robert.rejdak@umlub.pl; 2 Department of Analytical Chemistry, Medical University of Lublin, Chodźki 4A, 20-093 Lublin, Poland; malgorzatatatarczakmichalewska@umlub.pl; 3 Department of Water Supply and Wastewater Disposal, Lublin University of Technology, Nadbystrzycka 40B, 20-618 Lublin, Poland; b.kowalska@pollub.pl; 4 Department of Applied Mathematics, University of Technology, Nadbystrzycka 38D, 20-618 Lublin, Poland; majerek@gmail.com; 5 Chair and Department of Nephrology, Medical University of Lublin, Jaczewskiego 8, 20-090 Lublin, Poland; izabela.zakrocka@umlub.pl (I.Z.); wojciech.zaluska@umlub.pl (W.Z.)

**Keywords:** hemodialysis, aqueous humor, trace elements, cataract, ICP-MS

## Abstract

Numerous reports have proven that dialysis patients experience disturbances in the levels of elements in biological fluids. Disturbances in the homeostasis of essential elements or the appearance of highly toxic elements are serious problems also in clinical ophthalmology. The purpose of this study was to investigate the influence of hemodialysis (HD) on the elemental composition of anterior chamber aqueous humor (AH) in patients undergoing cataract surgery. The study involved 22 patients. The control group enrolled 16 patients (age 75.68 ± 9.67, female 54.55%, male 45.45%) with cataract and normal kidney function (control), and the second group included six patients (age 70.33 ± 12.74, female 33.33%, male 66.67%) with cataract undergoing HD treatment. The elements quantification was established using an inductively coupled plasma optical emission spectrometer (ICP-MS). In the eye fluid of dialysis patients, there were increased levels of manganese (Mn) and mercury (Hg) and decreased levels of vanadium (V) and zinc (Zn). In addition, a statistically significant increase in the Hg/Zn and Hg/selenium (Se) ratios and a lowering of the iron (Fe)/Mn ratio were observed in the studied group in comparison to the control. The obtained results indicated the need for Zn and Se supplementation in order to eliminate the hazards caused by Hg toxicity. A lower level of V in the eye fluid of dialysis patients may have a positive effect on maintaining a calcium and phosphorus homeostasis. Our study gives a deep insight into changes of elements concentrations in AH induced by HD.

## 1. Introduction

Cataract surgery is a procedure that can improve or even restore patients’ vision. However, complications such as vitreous hemorrhage (VH), retinal detachment, intraocular lens dislocation, dropped nucleus, and wound dehiscence may occur as a consequence of this procedure [1]. Many reports have demonstrated that, for patients with end-stage renal disease (ESRD), the risk of cataract [2], as well as postoperative complications, is significantly higher [3,4,5,6,7]. The first retrospective case–control study that undertook a risk assessment of cataract surgery in patients with ESRD was reported in 2020 [8]. The study covered 352 cases and 1760 controls who underwent cataract-removal surgery between 2002 and 2013 in Taiwan. Complications following cataract surgery were the main measures of the outcome. Patients with ESRD were found to be more likely to experience VH, reoperation for dropped nucleus or vitreous complications, and corneal edema within 3 months of cataract surgery.

The homeostasis of macro and microelements is ensured by the processes of absorption, storage, and excretion. These steps, however, are not universal and differ depending on the kind of trace elements and the degree of ionic valency [9]. Homeostasis is regulated mostly by absorption from the gastrointestinal tract and excretion through the urine. The majority of toxic elements are eliminated from the body by the kidneys. Due to the impaired glomerular filtration, metals are actively reabsorbed from the blood, causing systemic effects and further kidney damage [10,11,12,13]. In renal failure, in addition to causing a deficiency of essential elements, an accumulation of toxic elements may be expected as a result of reduced excretion, as well as the interdependent relationship between the elements. The concentrations of the elements in the population of dialyzed patients are determined mainly in the blood serum (aluminum (Al), silicon (Si), copper (Cu), selenium (Se), nickel (Ni), zinc (Zn), strontium (Sr), and chromium (Cr)). However, it should be emphasized that presented results differ depending on the dialysis center, which indicates the environmental aspect of bioaccumulation. The accumulation of Cr, vanadium (V), Ni, and Cu has been described in patients with chronic kidney disease (CKD) [14]. Children with CKD have increased levels of toxic elements such as cadmium (Cd) and lead (Pb) [9,15]. The levels of essential elements are also disturbed in CKD. Reduced levels of Zn, Se, Fe, and calcium (Ca) have been reported [16,17]. At some stage of CKD, nephrologists must consider renal replacement therapy (RRT) in the form of hemodialysis (HD) to purify the patient’s blood, remove water excess, and keep the electrolyte balance. Tonelli et al. [17] conducted a meta-analysis to summarize the trace element status of adult HD patients. The authors proved that the blood levels of Cd, Cr, Cu, Pb, and V were significantly higher, whereas Se, Zn, and Mn were lower in HD patients, concerning the controls. Thus, this kind of RRT can lead to a dangerous accumulation of toxic elements, as well as the deficiency of Zn and Se. The reasons for the observed changes result from possible contamination and insufficient elimination, as well as the excessive removal of essential trace elements. Without a doubt, some of them can be attributed to dialysis treatment, which causes not only the removal of waste products but, also, chemical contamination [12]. As it turns out, the contamination of dialysis fluids with trace metals from water or chemical concentrates, although extremely rare nowadays due to system control, cannot be ruled out [18].

The main ions, such as sodium (Na), potassium (K), and Ca, are routinely monitored in dialysis patients. The trace element levels have been determined mostly in the whole blood, serum, or plasma [17,18,19,20,21] of patients undergoing HD in comparison with healthy controls. However, some authors have suggested that elemental blood levels are not reliable markers. First of all, because many elements are bound to intracellular proteins [22] and, secondly, because of the compartmentalization of the trace elements [23]. Clinical studies have revealed the disturbances of the trace element levels in different tissues, blood, and urine. It was proven that strontium (Sr), molybdenum (Mo), Cd, and tin (Sn) may accumulate in tissues of the liver and bones of HD patients [24]. In the scalp hair of HD patients, the concentrations of beryllium (Be), arsenic (As), magnesium (Mg), Cr, Mn, Fe, Se, Mo, iodine (I), V, and cobalt (Co) were found to be significantly higher than those in healthy subjects [25]. The length of dialysis and dialysis efficacy, estimated through the Kt/V parameters, appeared to be not without significance for chronic exposure to these elements. The authors suggested that increased levels of Mg, Mn, Zn, and Se may be affected by these factors.

Disturbances in the homeostasis of essential elements or the appearance of highly toxic elements in AH are serious problems in clinical ophthalmology. The clinical significance of trace element accumulation, deficiency, or ratios is still the subject of much research and is not yet fully understood. Although the levels of macro- and microelements, both essential and toxic, in AH taken from cataract patients suffering from various coexisting diseases, like glaucoma, age-related macular degeneration (AMD), diabetes, retinopathy, and hypertension [26,27,28,29,30,31,32,33], were extensively investigated, the AH of cataract patients undergoing HD treatment has not yet been examined. The AH is considered sometimes as an ultrafiltrate of blood plasma [34]. However, there is evidence that some kind of differences can be found, especially in the concentration of ions, such as hydrogen carbonate (HCO_3_^−^), chloride (Cl^−^), Na^+^, and K^+^, which are higher in AH in comparison to plasma. The presence of these osmotically active elements is responsible for the osmotic pressure and the fluidity of the AH, which plays an important role in the nutrition of intraocular tissues [35]. It has been known since 1964 [36] that there is a link between the changes in intraocular pressure (IOP) and HD. IOP is believed to be caused by a decrease in plasma osmolality or a relative increase in the concentration of urea in the AH as a result of fluid displacement from the blood into the anterior chamber, due to the so-called “reverse urea effect” [37,38]. Nowadays, thanks to improved dialysis techniques, which ensure stable serum osmolality, the observed changes in IOP are much smaller [39]. In a recent meta-analysis by Chen et al., it was highlighted that the use of acetate dialysate can be responsible for IOP elevation, whereas dialysate substitution with bicarbonate prevents IOP variability [40]. Interestingly, Kalayci et al. reported in a study on 112 patients a mean decrease in IOP 1.4 ± 2 mm Hg after HD [41]. Dehydration and lowering body fluids’ osmolarity are considered the main mechanisms responsible for the IOP-lowering effect of HD.

Although, in some studies, no changes in ocular parameters following HD were shown, most researchers observed that HD modifies the ocular surface, visual acuity, IOP, retinal thickness, and refraction [42]. HD treatment may provoke pathologies of the cornea, conjunctiva, and lens and lead to retinal disturbances in the retrobulbar circulation [43,44,45,46,47]. Considering the changes that may occur in the eyes due to HD, comparative elemental studies are needed for the identification and evaluation of the risk of this kind of RRT. However, no one has yet tested the eye fluid in dialysis patients.

This study aimed to evaluate the influence of the HD treatment on the elemental composition of the anterior chamber fluid. All study participants underwent cataract surgery. The patients with ESRD who underwent HD were enrolled in an examined group, while the control group was constituted by patients without CKD.

## 2. Materials and Methods

### 2.1. Ethics Statement and Patients

The research material consisted of the AH from the anterior chamber of the eye taken from the subjects undergoing cataract surgery in the Chair and Department of General and Pediatric Ophthalmology, Medical University of Lublin, who were all Polish. The patients with ESRD were hemodialyzed three times a week in the Chair and Department of Nephrology, Medical University of Lublin, Poland. Each HD session lasted 4 h (12 h of HD a week for each patient). The study was approved by the Local Bioethical Committee of the Medical University of Lublin (approval no. KE-0254/245/2020). The work was carried out in adherence to the tenets of The Declaration of Helsinki. Of all patients undergoing surgery in 2020 to 2021, 6 HD patients with cataracts fulfilled the inclusion criteria (age 49.67 years; SD: 12.674, 8 females, 4 males), and 16 patients with cataracts (age 49.67 years; SD: 12.74, females 7, males 4) were matched to these patients. Sampling of the AH was done during surgical procedures, namely, cataract phacoemulsification. Aqueous fluid extraction was performed at the microscope through paracentesis at the first stage of the cataract removal procedure. A 27- or 30-gauge needle was used, with extraction of 0.1 to 0.2 mL of AH followed by injection of a balanced salt solution. After collection, the samples were immediately frozen and stored in 1.5-mL polypropylene tubes at −80 °C until analysis.

### 2.2. Sample Preparation Procedure

The wet mineralization of each sample (50–160 µL) was performed via the addition of 2 mL of 69% suprapur nitric acid, BAKER ANALYZED™ A.C.S. Reagent, J.T.Baker™, Thermo Fisher Scientific (Hampton, NH, USA), followed by heating to 180 °C in close Teflon containers in the microwave mineralization system TOPEX (Preekem, Shanghai, China). After mineralization, 0.5 mL of hydrochloric acid (Merck, Darmstadt, Germany) was added to stabilize some elements (As, Hg, Se, Mo, Tl, and Ag). Finally, the samples were diluted up to 10 mL by ultrapure water obtained from the purification system Milli-Q (Millipore, Darmstadt, Germany). The obtained results were recalculated, taking into account varied volumes of the AH sample, the volumes of mineralizates, and sample dilution factors. Thus, the collected data express the concentrations of elements in the real AH sample. The concentrations were expressed using analytical units common in trace analysis, such as parts-per-million (ppm, 10^−6^) and parts-per-billion (ppb, 10^−9^) (*m/v*).

### 2.3. Analytical Procedures

The inductively coupled plasma mass spectrometer Agilent 8900 ICP-MS Triple Quad (Agilent, Santa Clara, CA, USA) was employed for an elemental analysis. Most of elements were analyzed in helium (He) mode (5.5-mL/min helium flow); Se and As were analyzed in oxygen (O_2_) mode (gas O_2_, flow rate—30%). The plasma was used in general purpose mode with 1.550-kW RF power, the nebulizer gas flow was 1.07 L/min, the auxiliary gas flow was 0.9 L/min, and the plasma gas flow was 15 L/min. The acquisition time was from 0.1 to 2 s, depending on the predicted concentration of the element. ICP commercial analytical standards (Agilent Technologies, Santa Clara, CA, USA) were used for the calibration.

### 2.4. Statistical Analysis

The normality tests (the Shapiro–Wilk test) and tests of homogeneity of variance (the Levene test) were performed for the studied groups in the context of element concentrations [48,49,50]. In most cases, the normality and homogeneity of the variance did not meet the requirements. For intercorrelations, the Wilcoxon Mann–Whitney test was applied [51,52]. The effect sizes were evaluated by a biserial rank correlation coefficient [53]. The effect size interpretation was based on the literature data [54,55,56,57,58]. Statistical analyses were performed in the R statistical environment (R Core Team 2016) [59], using a number of libraries that extend the capabilities of the core version of the program [60,61,62,63].

## 3. Results and Discussion

### 3.1. Elemental Intercorrelations

**Figure 1:**
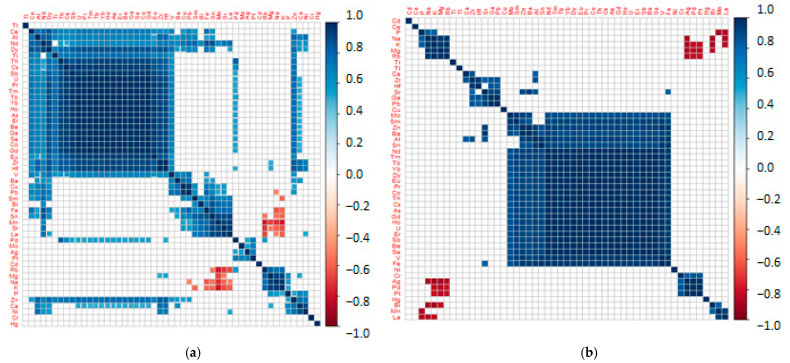
Correlation matrices prepared for the controls (**a**) and cases (**b**).

Inter-elemental correlation was conducted in both groups for all examined elements. Figure 1 presents the obtained correlation matrices. On the correlation matrices, the formation of clusters of the elements correlating with each other positively, negatively, or not correlated at all (red, blue, and white respectively) is observed. In the control group, there were distinct clusters of elements strongly correlated positively. The following red clusters can be distinguished: (V, Co, Be, gallium (Ga), As, Se, praseodymium (Pr), gadolinium (Gd), terbium (Tb), holmium (Ho), erbium (Er), thulium (Tm), ytterbium (Yb), uranium (U), antimony (Sb), cesium (Cs), thorium (Th), europium (Eu), hafnium (Hf), zirconium (Zr), titanium (Ti), dysprosium (Dy), neodymium (Nd), Al, and cerium (Ce0); Pb, Cu, and barium (Ba); lanthanum (La), Sr, Mn, Sn, Fe, bismuth (Bi), and samarium (Sm); silver (Ag), Mo, platinum (Pt), and palladium (Pd); phosphorus (P), K, Na, Mg, and rubidium (Rb); and Ni, Ca, Zn, and P. The strength of the negative correlations is not significant. In the group of cataract patients undergoing HD, the clusters, which are strongly positively correlated with each other, are reduced to the following: Fe, V, Co, Be, As, Se, Sb, Cs, Pr, Nd, Eu, Gd, Tb, Dy, Ho, Er, Tm, Yb, Th, U, Sn, Al, Sm, Mo, Ba, and Zn; La, Mn, and Bi; Pt, Pd, Ag, and Cr; Hf, Zr, Pb, and Ga; and K, Na, Rb, and Mg. Additionally, there are stronger negative correlations, i.e., P/La and Mn; La/Na and K; Bi/Mg; Bi/Na and K; Ag/P, Mg, Rb, Na, and K; and Pt, Pb/Mg, Rb, and K. In the group of negative correlations, it is noteworthy that toxic elements such as Bi and Pb affect the levels of the essential elements, such as Na, K, and Mg. Therefore, this confirms the belief that these toxic elements disturb the homeostasis and the absorption of key macronutrients. It turns out that noble metals (Ag and Pt) and some metals from the lanthanide group may also play such a role. The above dependencies in patients undergoing dialysis have not been reported so far.

### 3.2. Statistically Significant Differences between Cases and Controls

In most cases, there are no significant differences between the concentrations of the elements in both groups. The exceptions are statistically significant elevated levels of Cs, Eu, Hf, Nd, Rb, V, and Zr in the control group and a higher level of Hg and Mn in the cases group, assuming the significance level at α = 0.1. The descriptive statistics of the studied groups are summarized in Table 1. The results of the statistically significant differences between the patient groups using the Wilcoxon Mann–Whitney test are collected in Table 2.

**Figure 2:**
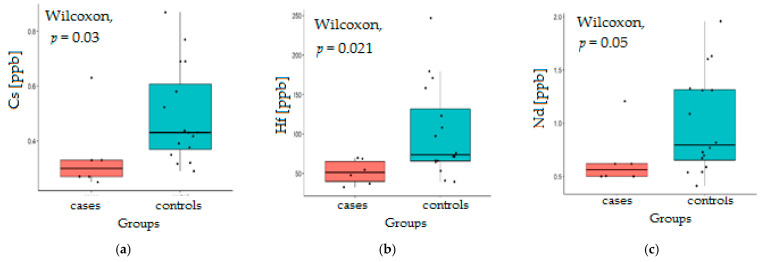

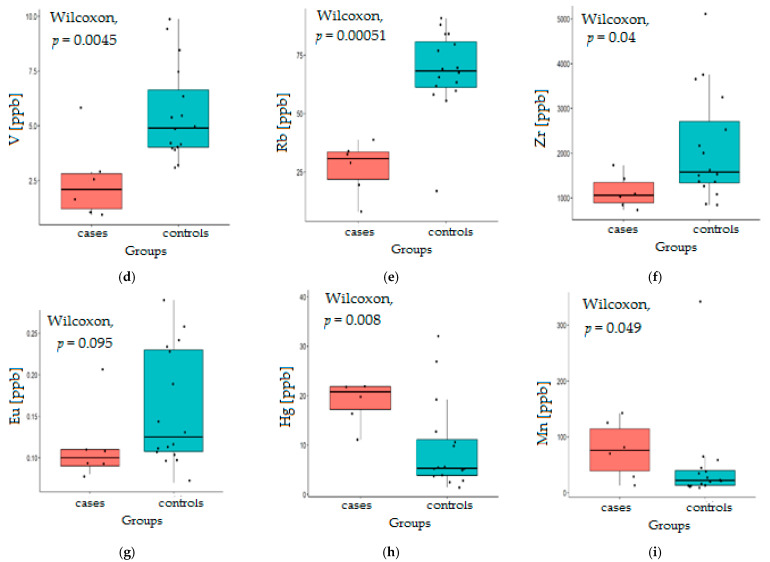
Statistical significance of the differences of the metallic contents in AH of HD patients (cases) compared to the controls for Cs (ppb) (**a**), Hf (ppb) (**b**), Nd (ppb) (**c**), V (ppb) (**d**), Rb (ppb) (**e**), Zr (ppb) (**f**), Eu (ppb) (**g**), Hg (ppb) (**h**), Mn (ppb) (**i**).

Among the elements whose concentration decreases in a statistically significant manner in the ocular fluid after HD is cesium (Cs). The accumulation of this element in the eye fluid of patients undergoing cataract surgery was demonstrated in a previous work [31]. Health hazards due to Cs accumulation arise, among others, from the chemical similarity of Cs to K. Serious heart problems, loss of consciousness, convulsions, and electrolyte disturbances (Na/K imbalance) have been associated with Cs overdose. Lowering the Cs level after HD (Figure 2a) seems to be a beneficial effect. If possible, this observation should be confirmed on a larger group of patients.

Based on the hierarchical grouping presented in a previous work [31], five clusters were created, taking into account the homogeneity of variance for individual elements, which were quantified in the eye fluid of patients undergoing cataract surgery. Taking into account the concentrations of the elements, the clusters were ranked in the following order: cluster 3 < cluster 1 < cluster 4 < cluster 2 < cluster 5.

The present study showed that some of the highest concentration elements in cluster number 5, such as the rare earth elements Eu, Nd, and V belonging to the transition metals, are eliminated during dialysis (Figure 2c,d,g). While the health importance of lanthanides is not widely acknowledged, a few reports have described the detection of V in the form of vanadium oxide (V_2_O_3_) in human endo- and epithelial lung cells or in the bones [64]. It is known that V is an element necessary for metabolic processes, including phospholipids, cholesterol and triglyceride metabolism, hepatic glucose oxidation, and glycogen synthesis [65]. However, excessive concentration of this metal causes irreversible damage to various tissues and organs, including the liver and kidneys. V is easily taken up in the gastrointestinal tract [66]. Vanadate (H_2_VO_4_^−^) is partially reduced in the stomach and precipitated in the form of VO(OH)_2_ in the slightly alkaline medium of the intestines. V can also enter the bloodstream by injection or infusion when present as a ‘contaminant’ in infusion solutions [67]. The dominant forms of V found in the blood are vanadate (V) and vanadyl (IV). These forms are stabilized by interactions with a plasma protein, namely transferrin (65%). The action of V in the water compartment is related to, among others, phosphate–V antagonism. This similarity causes phosphatase-dependent enzyme inhibition and kinases activation [9]. Based on the results obtained, it can be concluded that HD prevents a dangerous accumulation of V in the eye fluid.

Zr, previously classified as cluster 1, also undergoes a statistically significant elimination during dialysis. This is definitely a positive effect, taking into account the toxicity of this element, especially for the respiratory system, and the possibility of immunization. Zr compounds are commonly added to cosmetics, and so far, only a few countries, such as the USA and Canada, qualify them as non-recommended ingredients.

Additionally, a significant decrease in Hf and Rb was observed in dialysis patients (Figure 2b,e). However, it seems dangerous that the levels of highly toxic elements such as Mn and Hg not only do not change but even increase statistically in the AH of dialysis patients (Figure 2h,i). Mn belongs to the group of essential trace elements, as it is a cofactor of enzymes, i.e., arginase and superoxide dismutase [22]. The level of Mn in whole blood was set at 10–12 ppb. Exposure to environmental pollution, particularly dust and fumes, can cause excessive Mn toxicity known as manganism. The symptoms of this disease resemble those associated with Parkinson’s disease and trigger impaired motor skills and cognitive disorders [23]. Mn in water is especially dangerous because of its higher bioavailability compared to other dietary sources. Due to the Mn content in high-purity water, which is 0.703 ppb, the dialysis feed water may be a potential source of Mn contamination, which may accumulate due to high exposure during the HD procedure [5]. Since the volumes of dialysate to which patients are exposed exceed 300 L/week [17], even small amounts of toxic substances can lead to their accumulation in various tissues and body fluids. In our study, the median Mn concentration in AH of HD patients with cataracts was 75.839 ppb and was statistically significantly higher (*p* = 0.051) compared to the control (21.919 ppb).

Ultrastructural changes in the kidneys progress as a result of aging and stress, which can also include exposure to toxic substances. An important environmental toxin that has a nephrotoxic effect is Hg. It is known that exposure to Hg ions leads to glomerular and tubular damage. It has been shown that older adults with renal failure are more sensitive to Hg [68]. A previous study [31] measured the levels of chemical elements in the AH of patients undergoing cataract surgery. The pilot study included 115 patients. In this test, fairly high levels of Hg, ranging from 0 to 4.303 ppm, were detected. In the present study, it was found that there is a statistically significant increase in the Hg level in HD patients compared to the controls (*p* = 0.011). In the control group, Hg ranged from 1.417 to 32.018 ppb, while, in the group of cataract dialysis patients, the concentration range was from 11.077 to 39.5 ppb.

### 3.3. Relationships between Elements’ Ratios

Exposure to Cd has been shown to be related to renal dysfunction [69,70,71]. The symptoms that accompany renal tubular damage are proteinuria and hypercalciuria [72]. In severe conditions, complications such as glucosuria, aminoaciduria, hyperphosphaturia, polyuria, and reduced buffering capacity may occur [73]. The Cd-induced release of calcium from bone tissue may affect the skeletal system through its demineralization [74]. Cd can accumulate in tissues for a long time due to its long half-life (30 years). The half-life of Cd in the blood (three to four months) is short and is indicative of the current exposure [69]. There were no differences in the level of this element in the eye fluid of both studied groups. However, a more reliable comparison, allowing the detection of a possible hazard, is the comparison of the ratios of the elements that affect the bioavailability of Cd. As can be seen in Table 3, there were no statistically significant differences in the ratios of Cd to Ca, Cu, Fe, P, Se, and Zn in both patient groups. However, this did not prove the Cd safety level in patients suffering from ESRD but, rather, the effectiveness of HD treatment with the aim of balancing the levels of compared elements. Although, nowadays, many countries report decreased exposure to Cd [75], due to its long half-life, future studies should be conducted on the bigger cohorts.

**Figure 3:**
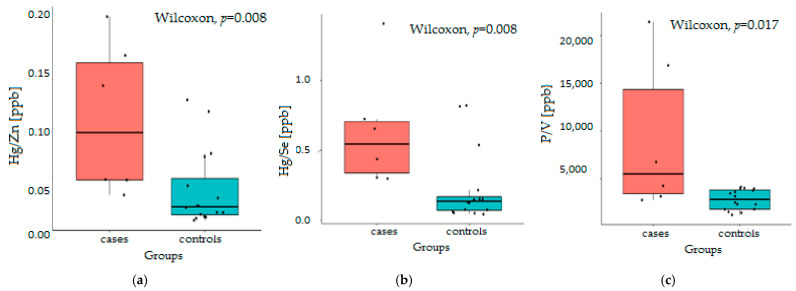
Differences in the Hg/Zn (**a**), Hg/Se (**b**), and P/V (**c**) ratios in AH of HD patients compared to the control.

Other toxic elements, such as Pb or Cd, affect the bioavailability of calcium and phosphorus, impeding proper bone mineralization. In turn, Hg works by attaching to proteins and blocking enzymes important for life. It has been proven that Hg complexes readily with cysteine residues, supplanting Zn and reducing its reabsorption [76]. Large amounts of Hg come from amalgam fillings; seafood; fish (salmon, tuna, and swordfish); coal incinerators; paints; and fluorescent bulbs. The rations of Hg/Se and Hg/Zn differ significantly in both groups, with *p* < 0.02. They are higher in the case group, suggesting not only an elevated level of Hg in the eye fluid of HD patients but, also, insufficient levels of Zn and Se (Figure 3). Metal ion transporters like divalent metal ion transporter-1(DMT-1) or ZIP-8 are nonselective for divalent metal ions. Their Fe- or Zn-binding preferences change when exposed to Cu, Mn, Co, Ni, Pb, and Cd. Thus, the low level of Fe is nonbeneficial, as it induces the overexpression of transporters, increasing the uptake of toxic metals. Our study found that dialysis patients have an underestimated Fe/Mn ratio. This result may be influenced by a low Fe level, insufficient to prevent Mn uptake.

In 1972, Summers and Moran presented the original hypothesis that V may influence Ca and P metabolism [77]. The residence time of V in the bones, where it replaces P in the hydroxyapatite Ca_5_(PO_4_)_3_OH, is about one month [70,78]. In our experiments, the P/V ratio is significantly higher in HD patients, which is the result of lowering the level of V in the eye fluid but, also, indicates that the HD processes did not worsen this ratio.

Summarizing, the obtained results revealed substantial variations in the elemental compositions between the HD group in comparison to the control group with normal kidney functions. However, only a relatively small number of elements exhibited statistically significant variations. The cataract patients in the HD group had significant differences in the levels of Mn, Hg, Zn, Eu, Rb, V, Cs, Hf, and Nd and the ratios of Hg/Zn, Hg/Se, P/V, and Fe/Mn in comparison to the control group (*p* < 0.05). It turned out that, in the eye fluid of dialysis patients, there were increased levels of Mn and Hg and decreased levels of V and Zn. A statistically significant increase in the interrelationships between Hg/Zn and Hg/Se was observed, suggesting the need for Zn and Se supplementation in order to eliminate the hazards caused by Hg toxicity. The increased level of Mn influenced the Fe/Mn ratio, which was significantly lower in patients undergoing HD. HD does not change the systemic status of Fe and does not eliminate Mn from the eye. The supplementation of dialysis patients with Fe can affect the elimination of Mn. This study showed a statistically significant lower level of V in the eye fluid of dialysis patients, which may have a positive effect on maintaining the Ca and P homeostasis. However, any final conclusions regarding the supplementation of patients undergoing HD require further studies on larger groups of patients. We cannot exclude differences in the elemental composition of AH determined by other research groups, because the accumulation of toxic metals is strongly connected with the environment. The limitations of the above study cover the possible contamination of dialysis fluids as a source of trace metals responsible for patient poisoning.

Our study may have practical implications. Since micronutrients deficiency is an essential complication of RRT, especially in continuous methods, to modify element contents in body fluids, a change in the RRT mode can be helpful. In a study described by Wu et al. [79], it was shown that double-filtration or dialysate-recycling methods may significantly reduce the loss of some elements, like Cu or Zn. Another technical issue related to the RRT and body fluid compositions was raised by Bogye et al. [80]. In a study on 28 hemodialyzed patients, the loss of selenium through polysulfone membrane pores was suggested, indicating the role of different types of membranes in element-level disturbances. Additionally, the Se deficiency in HD patients was shown to not be related to their nutritional status but, rather, with the form of RRT, indicating that food supplementation may have a lower impact on element compositions in body fluids than the HD parameters [81]. Another possible method to modify AH element concentrations is the administration of eye drops containing deficient agents, as reported by Wang et al. in a study with zinc diethyldithiocarbamate [82]. Since Se and Zn are well-known antioxidants, their deficiencies in AH may have a tremendous impact on cataract surgery outcomes. Despite that we did not observe any complications in the analyzed groups, further studies are needed to clarify this aspect of cataract treatment in HD patients. The conducted research showed that the compensation of deficiencies of elements such as Zn, Fe, and Se by supplementation or, especially, the modification of the hemodialysis mode may have one more benefit in the form of the elimination of Mn and Hg. Our observations require confirmation in new groups recruited among patients of nephrology and ophthalmology clinics who meet the inclusion and exclusion criteria.

## 4. Conclusions

The first step in understanding trace element metabolism and its role in the pathogenesis of diseases is to establish their distribution in different compartments, identifying the sites of accumulation, deficiency of trace elements, or disturbances in the element ratios. Since active secretion represents an important part of AH production from the ciliary epithelium, besides diffusion and ultrafiltration, it should be considered as an independent compartment. Many published reports have shown that dialysis patients experience disturbances in the levels of trace elements in biological fluids and tissues. This work presented, for the first time, the elemental composition of the anterior chamber fluid in cataract patients undergoing HD. The study identified a significant factor that poses a threat to the human eye, which is the increased level of Hg and Mn in patients undergoing HD. However, it should be emphasized that HD also has a beneficial effect on the elemental composition of the eye fluid. A significant decrease in Cs and V was detected, which had a positive effect on the Ca–P and Na–K balances of the body fluids.

## Figures and Tables

**Table 1:** Descriptive statistics of the studied groups of patients undergoing cataract surgery: the case group of HD patients undergoing hemodialysis (*n* = 6) and the control group of patients without hemodialysis (*n* = 16). Elemental mean concentrations expressed in µg L^−1^ are presented, together with spread measurements such as the variance coefficient (cv), standard deviation (sd), the first quartile (*q*_1_), and the third quartile (*q*_3_).

Variable	Group	Min	Max	Median	*q*_1_	*q*_3_	Mean	sd	cv
Cs	control	0.29	0.87	0.43	0.369	0.607	0.493	0.177	35.9
	case	0.25	0.63	0.3	0.27	0.33	0.347	0.143	41.21
Eu	control	0.07	0.29	0.125	0.108	0.23	0.158	0.07	44.3
	case	0.08	0.21	0.1	0.09	0.11	0.115	0.048	41.74
Hf	control	39.559	246.908	73.745	65.628	131.714	102.083	58.829	57.63
	case	32.367	69.748	51.287	39.848	65.169	51.753	15.635	30.21
Nd	control	0.41	1.958	0.795	0.651	1.314	1	0.468	46.8
	case	0.5	1.207	0.562	0.501	0.62	0.658	0.275	41.79
Rb	control	16.852	90.826	68.346	61.255	80.744	68.181	17.633	25.86
	case	8.05	38.819	30.752	21.876	33.54	26.96	11.283	41.85
V	control	3.093	9.873	4.906	4.022	6.636	5.55	2.161	38.94
	case	0.93	5.822	2.093	1.209	2.81	2.483	1.814	73.06
Zn	control	65.368	825.881	240.597	178.358	444.86	317.323	209.009	65.87
	case	102.284	659.523	252.261	142.43	320.648	285.932	204.407	71.49
Mn	control	8.88	341.835	21.919	13.149	39.837	46.73	80.514	172.3
	case	13.282	142.479	75.839	39.219	114.46	76.964	51.141	66.45
Hg	control	1.417	32.018	5.307	3.846	11.114	9.485	9.063	95.55
	case	11.077	39.5	20.761	17.212	21.855	21.725	9.612	44.24

**Table 2:** Results of statistically significant differences between the patient groups enrolled in the study (*n* = 22) in the nonparametric Mann–Whitney *U* test. Abbreviations: r—the rank-biserial correlation, CI—a confidence interval, and *p*—the probability value (*p*-value).

Element	*p*	log_e_ *W_(_*_Mann–Whitney)_	r	CI_95%_
Cs	0.030	4.36	0.62	0.18; 0.96
Eu	0.095	4.26	0.48	0.04; 0.94
Hf	0.025	4.37	0.65	0.30; 0.96
Nd	0.050	4.32	0.56	0.16; 0.94
Rb	0.002	4.51	0.90	0.62; 1.00
V	0.007	4.44	0.77	0.25; 1.00
Zr	0.043	4.33	0.58	0.20; 0.90
Mn	0.051	3.04	−0.56	−1.00; −0.16
Hg	0.011	2.56	−0.73	−0.92; −0.28

**Table 3:** Differences between the control (group I, *n* = 16) and HD patients (group 2, *n* = 6) considering the selected elemental ratios. Statistically significant correlations are marked in red.

Element Ratios	Group 1	Group 2	n1	n2	Statistic	*p*
cadmium elimination						
Cd/Ca	control	case	16	6	37	0.449
Cd/Cu	control	case	16	6	44	0.802
Cd/Fe	control	case	16	6	45	0.858
Cd/P	control	case	16	6	41	0.641
Cd/Se	control	case	16	6	38	0.494
Cd/Zn	control	case	16	6	54	0.693
iron balancing						
Fe/Co	control	case	16	6	39	0.541
Fe/Cu	control	case	16	6	50	0.914
Fe/Mn	control	case	16	6	82	0.010
Fe/Ni	control	case	16	6	36	0.407
phosphorous balancing						
P/Fe	control	case	16	6	46	0.914
P/Mg	control	case	16	6	26	0.115
P/V	control	case	16	6	16	0.017
P/Ca	control	case	16	6	36	0.407
sodium/potassium balancing						
Na/K	control	case	16	6	64	0.261
mercury elimination						
Hg/Se	control	case	16	6	13	0.008
Hg/Zn	control	case	16	6	13	0.008
lead, strontium elimination						
Pb/Ca	control	case	16	6	50	0.914
Pb/Cu	control	case	16	6	51	0.858
Pb/Fe	control	case	16	6	47	0.971
Pb/Se	control	case	16	6	45	0.858
Pb/Zn	control	case	16	6	49	0.971
Sr/Ca	control	case	16	6	31	0.231

## Data Availability

The data presented in this study are available upon request from Joanna Dolar-Szczasny.

## References

[1] Stein J.D., Grossman D.S., Mundy K.M., Sugar A., Sloan F.A. (2011). Severe adverse events after cataract surgery among medicare beneficiaries. Ophthalmology.

[2] Liu Y.T., Hung T.Y., Lee Y.K., Huang M.Y., Hsu C.Y., Su Y.C. (2017). Association between Chronic Kidney Disease and Risk of Cataract: A Nationwide Retrospective Cohort Study. Am. J. Nephrol..

[3] You T.H., Sidaoui J., Marone L.K., Avgerinos E.D., Makaroun M.S., Chaer R.A. (2014). Limited survival in dialysis patients undergoing intact abdominal aortic aneurysm repair. J. Vasc. Surg..

[4] Hansen L.S., Hjortdal V.E., Andreasen J.J., Mortensen P.E., Jakobsen C.J. (2015). 30-day mortality after coronary artery bypass grafting and valve surgery has greatly improved over the last decade, but the 1-year mortality remains constant. Ann. Card. Anaesth..

[5] Gajdos C., Hawn M.T., Kile D., Robinson T.N., Henderson W.G. (2013). Risk of major nonemergent inpatient general surgical procedures in patients on long-term dialysis. JAMA Surg..

[6] Dursun D., Akova Y.A., Akman A., Oto S., Aydin P. (2000). Complications of extracapsular cataract surgery in chronic renal failure patients. Eye.

[7] Luo L.H., Xiong S.H., Wang Y.L. (2015). Results of cataract surgery in renal transplantation and hemodialysis patients. Int. J. Ophthalmol..

[8] Hsiao C.H., Liang F.W., Ho C.H., Chen Y.C., Wang J.J., Hsing C.H., Wu C.C. (2020). Cataract surgery-related complications in patients with end-stage renal disease- a nationwide population-based study in Taiwan. Sci. Rep..

[9] Filler G., Felder S. (2014). Trace elements in dialysis. Pediatr. Nephrol..

[10] Meliker J.R., Wahl R.L., Cameron L.L., Nriagu J.O. (2007). Arsenic in drinking water and cerebrovascular disease, diabetes mellitus, and kidney disease in Michigan: A standardized mortality ratio analysis. Environ. Health.

[11] Soderland P., Lovekar S., Weiner D.E., Brooks D.R., Kaufman J.S. (2010). Chronic kidney disease associated with environmental toxins and exposures. Adv. Chronic Kidney Dis..

[12] D’Haese P.C., De Broe M.E. (1996). Adequacy of dialysis: Trace elements in dialysis fluids. Nephrol. Dial. Transplant..

[13] Filler G., Roach E., Yasin A., Sharma A.P., Blake P.G., Yang L. (2012). High prevalence of elevated lead levels in pediatric dialysis patients. Pediatr. Nephrol..

[14] Prodanchuk M., Makarov O., Pisarev E., Sheiman B., Kulyzkiy M. (2014). Disturbances of trace element metabolism in ESRD patients receiving hemodialysis and hemodiafiltration. Cent. Eur. J. Urol..

[15] Wihelm M., Hanewinckel B., Bläker F. (1986). Influence of haemodialysis and renal transplantation on trace element concentrations in children with chronic renal failure. Eur. J. Pediatrics.

[16] Gallieni M., Brancaccio D., Cozzolino M., Sabbioni E. (1996). Trace elements in renal failure: Are they clinically important?. Nephrol. Dial. Transplant..

[17] Tonelli M., Wiebe N., Hemmelgarn B., Klarenbach S., Field C., Manns B., Thadhani R., Gill J. (2009). Alberta Kidney Disease Network. Trace elements in hemodialysis patients: A systematic review and meta-analysis. BMC Med..

[18] Greenberg K.I., Choi M.J. (2021). Hemodialysis Emergencies: Core Curriculum 2021. Am. J. Kidney Dis..

[19] Gómez de Oña C., Martínez-Morillo E., Gago González E., Vidau Argüelles P., Fernández Merayo C., Álvarez Menéndez F.V. (2016). Variation of trace element concentrations in patients undergoing hemodialysis in the north of Spain. Scand. J. Clin. Lab. Investig..

[20] Tonelli M., Wiebe N., Bello A., Field C.J., Gill J.S., Hemmelgarn B.R., Holmes D.T., Jindal K., Klarenbach S.W., Manns B.J. (2017). Alberta Kidney Disease Network. Concentrations of Trace Elements in Hemodialysis Patients: A Prospective Cohort Study. Am. J. Kidney Dis..

[21] Tonelli M., Wiebe N., Bello A., Field C.J., Gill J.S., Hemmelgarn B.R., Holmes D.T., Jindal K., Klarenbach S.W., Manns B.J. (2018). Alberta Kidney Disease Network. Concentrations of Trace Elements and Clinical Outcomes in Hemodialysis Patients: A Prospective Cohort Study. Clin. J. Am. Soc. Nephrol..

[22] Hambidge M. (2003). Biomarkers of trace mineral intake and status. J. Nutr..

[23] Krachler M., Prohaska T., Koellensperger G., Rossipal E., Stingeder G. (2000). Concentrations of selected trace elements in human milk and in infant formulas determined by magnetic sector field inductively coupled plasma-mass spectrometry. Biol. Trace Elem. Res..

[24] Alfrey A.C., Smythe W.R., Drukker W., Parsons F.M., Maher J.F. (1983). Trace Metals and Regular Dialysis. Replacement of Renal Function by Dialysis.

[25] Ochi A., Ishimura E., Tsujimoto Y., Kakiya R., Tabata T., Mori K., Shoji T., Yasuda H., Nishizawa Y., Inaba M. (2011). Trace elements in the hair of hemodialysis patients. Biol. Trace Elem. Res..

[26] Kayiklik A., Alyamac Sukgen E. (2019). Biochemical analysis of aqueous humor in diabetic and non-diabetic patients with cataracts. Ophthalmol. J..

[27] Aydin E., Cumurcu T., Özugurlu F., Özyurt H., Sahinoglu S., Mendil D., Hasdemir E. (2005). Levels of Iron, Zinc, and Copper in Aqueous Humor, Lens, and Serum in Nondiabetic and Diabetic Patients: Their Relation to Cataract. Biol. Trace Elem. Res..

[28] Stopa P., Rejdak R., Michalke B., Chaudhri A., Schlotzer-Schrehardt U., Kruse F.E., Zrenner E., Junemann A.G. (2010). Levels of Aqueous Humour Trace Elements in Patients With Age-Related Macular Degeneration. Investig. Ophthalmol. Vis. Sci..

[29] Jünemann A.G., Stopa P., Michalke B., Chaudhri A., Reulbach U., Huchzermeyer C., Schlötzer-Schrehardt U., Kruse F.E., Zrenner E., Rejdak R. (2013). Levels of aqueous humor trace elements in patients with non-exsudative age-related macular degeneration: A case-control study. PLoS ONE.

[30] Hohberger B., Chaudhri M.A., Michalke B., Lucio M., Nowomiejska K., Schlötzer-Schrehardt U., Grieb P., Rejdak R., Jünemann A.G.M. (2018). Levels of aqueous humor trace elements in patients with open-angle glaucoma. J. Trace Elem. Med. Biol..

[31] Dolar-Szczasny J., Święch A., Flieger J., Tatarczak-Michalewska M., Niedzielski P., Proch J., Majerek D., Kawka J., Mackiewicz J. (2019). Levels of Trace Elements in the Aqueous Humor of Cataract Patients Measured by the Inductively Coupled Plasma Optical Emission Spectrometry. Molecules.

[32] Schmeling M., Gaynes B.I., Tidow-Kebritchi S. (2014). Heavy metal analysis in lens and aqueous humor of cataract patients by total reflection X-ray fluorescence spectrometry. Powder Diffr..

[33] Flieger J., Dolar-Szczasny J., Rejdak R., Majerek D., TatarczakMichalewska M., Proch J., Blicharska E., Flieger W., Baj J., Niedzielski P. (2021). The Multi-Elemental Composition of the Aqueous Humor of Patients Undergoing Cataract Surgery, Suffering from Coexisting Diabetes, Hypertension, or Diabetic Retinopathy. Int. J. Mol. Sci..

[34] Gelatt K.N. (2007). Veterinary Ophthalmology.

[35] Ergun D.D., Dursun S., Ergun S., Ozcelik D. (2021). The Association between Trace Elements and Osmolality in Plasma and Aqueous Humor Fluid in Diabetic Rabbits. Biol. Trace Elem. Res..

[36] Sitprija V., Holmes J.H., Ellis P.P. (1964). Changes in intraocular pressure during hemodialysis. Investig. Ophthalmol..

[37] William J.H., Gilbert A.L., Rosas S.E. (2015). Keeping an eye on dialysis: The association of hemodialysis with intraocular hypertension. Clin. Nephrol..

[38] Levy J., Tovbin D., Lifshitz T., Zlotnik M., Tessler Z. (2005). Intraocular pressure during haemodialysis: A review. Eye.

[39] Chelala E., Dirani A., Fadlallah A., Slim E., Abdelmassih Y., Fakhoury H., Baz P., Bejjani R. (2015). Effect of hemodialysis on visual acuity, intraocular pressure, and macular thickness in patients with chronic kidney disease. Clin. Ophthalmol..

[40] Chen S.-H., Lu D.-W., Ku W.-C., Chuang L.-H., Ferng S.-H., Chen Y.-J., Lu Y.-H., Chai P.Y.-C. (2021). Changes in Intraocular Pressure during Hemodialysis: A Meta-analysis. J. Glaucoma.

[41] Kalayci M., Hassan I.A., Keinan I.A., Cetinkaya E., Suren E., Tahtabasi M., Sumbul H.E. (2020). The Effect of Hemodialysis on Axial Length, Ocular Surface, and Intraocular Pressure in Patients with End-Stage Renal Failure. Int. J. Gen. Med..

[42] Vrabec R., Vatavuk Z., Pavlović D., Sesar A., Cala S., Mandić K., Bućan K. (2005). Ocular findings in patients with chronic renal failure undergoing haemodialysis. Coll. Antropol..

[43] Diaz-Couchoud P., Bordas F.D., Garcia J.R., Camps E.M., Carceller A. (2001). Corneal disease in patients with chronic renal insufficiency undergoing hemodialysis. Cornea.

[44] Charlton J.F., Schwab I.R., Stuchell R. (1996). Tear hyperosmolarity in renal dialysis patients asymptomatic for dry eye. Cornea.

[45] Niutta A., Spicci D., Barcaroli I. (1993). Fluoroangiographic findings in hemodialyzed patients. Ann. Ophthalmol..

[46] Tosun O., Davutluoglu B., Arda K., Boran M., Yarangumeli A., Kurt A., Ozkan D. (2007). Determination of the effect of a single hemodialysis session on retrobulbar blood hemodynamics by color Doppler ultrasonography. Acta Radiol..

[47] Mullaem G., Rosner M.H. (2012). Ocular problems in the patient with end-stage renal disease. Semin. Dial..

[48] Razali N., Wah Y. (2011). Power Comparisons of Shapiro-Wilk, Kolmogorov-Smirnov, Lilliefors and Anderson-Darling tests. J. Stat. Modeling Anal. (JOSMA).

[49] Shapiro S.S., Wilk M.B. (1965). An analysis of variance test for normality (complete samples). Biometrika.

[50] Brown M.B., Forsythe A.B. (1974). Robust Tests for the Equality of Variances. J. Am. Stat. Assoc..

[51] Fay M.P., Proschan M.A. (2010). Wilcoxon-Mann-Whitney or t-test? On assumptions for hypothesis tests and multiple interpretations of decision rules. Stat. Surv..

[52] Neuhäuser M. (2011). Wilcoxon–Mann–Whitney Test.

[53] Cureton E.E. (1956). Rank-biserial correlation. Psychometrika.

[54] Cohen J. (1992). A power primer. Psychol Bull..

[55] Cohen J. (2013). Statistical Power Analysis for the Behavioral Sciences.

[56] Hemphill J.F. (2003). Interpreting the magnitudes of correlation coefficients. Am. Psychol..

[57] Gignac G., Szodorai E. (2016). Effect size guidelines for individual differences researchers. Personal. Individ. Differ..

[58] Funder D.C., Ozer D.J. (2019). Evaluating effect size in psychological research: Sense and nonsense. Adv. Methods Pract. Psychol. Sci..

[59] R Core Team (2018). R: A Language and Environment for Statistical Computing.

[60] Wickham H. (2016). Ggplot2: Elegant Graphics for Data Analysis.

[61] Patil I. (2021). Visualizations with statistical details: The ggstatsplot approach. J. Open Source Softw..

[62] Kassambara A. (2021). Rstatix: Pipe-Friendly Framework for Basic Statistical Tests. https://CRAN.R-project.org/package=rstatix.

[63] Sjoberg D.D., Curry M., Hannum M., Larmarange J., Whiting K., Zabor E.C., Bai X., Drill E., Flynn J., Hannum M. (2021). Gtsummary: Presentation-Ready Data Summary and Analytic Result Tables. https://CRAN.R-project.org/package=gtsummary.

[64] Wörle-Knirsch J.M., Kern K., Schleh C., Adelhelm C., Feldmann C., Krug H.F. (2007). Nanoparticulate Vanadium Oxide Potentiated Vanadium Toxicity in Human Lung Cells. Environ. Sci. Technol..

[65] Barrio D.A., Etcheverry S.B. (2006). Vanadium and bone development: Putative signaling pathways. Can. J. Physiol. Pharmacol..

[66] Wilk A., Szypulska-Koziarska D., Wiszniewska B. (2017). The toxicity of vanadium on gastrointestinal, urinary and reproductive system, and its influence on fertility and fetuses malformations. Postepy Hig. Med. Dosw..

[67] Bridges C.C., Zalups R.K. (2017). The aging kidney and the nephrotoxic effects of mercury. J. Toxicol. Environ. Health B Crit. Rev..

[68] Järup L. (2002). Cadmium overload and toxicity. Nephrol. Dial. Transplant..

[69] Fevrier-Paul A., Soyibo A.K., Mitchell S., Voutchkov M. (2018). Role of Toxic Elements in Chronic Kidney Disease. J. Health Pollut..

[70] Rafati Rahimzadeh M., Rafati Rahimzadeh M., Kazemi S., Moghadamnia A.A. (2017). Cadmium toxicity and treatment: An update. Casp. J. Intern. Med..

[71] Patrick L. (2003). Toxic metals and antioxidants: Part II. The role of antioxidants in arsenic and cadmium toxicity. Altern. Med. Rev..

[72] Gonick H.C. (2008). Nephrotoxicity of cadmium & lead. Indian J. Med. Res..

[73] Staessen J.A., Roels H.A., Emelianov D., Kuznetsova T., Thijs L., Vangronsveld J., Fagard R. (1999). Environmental exposure to cadmium, forearm bone density, and risk of fractures: A prospective population study. Public Health and Environmental Exposure to Cadmium (PheeCad) Study Group. Lancet.

[74] Nordberg G.F. (2004). Cadmium and health in the 21st century--historical remarks and trends for the future. Biometals.

[75] Keil D.E., Berger-Ritchie J., McMillin G.A. (2011). Testing for Toxic Elements: A Focus on Arsenic, Cadmium, Lead, and Mercury. Lab. Med..

[76] Summers J.D., Moran E.T. (1972). Interaction of dietary vanadium, calcium and phosphorus for the growing chicken. Poult. Sci..

[77] Heinemann G., Fichtl B., Vogt W. (2003). Pharmacokinetics of vanadium in humans after intravenous administration of a vanadium containing albumin solution. Br. J. Clin. Pharmacol..

[78] Thompson K.H., Lichter J., LeBel C., Scaife M.C., McNeill J.H., Orvig C. (2009). Vanadium treatment of type 2 diabetes: A view to the future. J. Inorg. Biochem..

[79] Wu B., Ji D., Xu B., Fan R., Gong D. (2019). New modes of continuous renal replacement therapy using a refiltering technique to reduce micronutrient loss. Hemodial. Int..

[80] Bogye G., Tompos G., Alfthan G. (2000). Selenium depletion in hemodialysis patients treated with polysulfone membranes. Nephron.

[81] Dworkin B., Weseley S., Rosenthal W.S., Schwartz E.M., Weiss L. (1987). Diminished blood selenium levels in renal failure patients on dialysis: Correlations with nutritional status. Am. J. Med. Sci..

[82] Wang S., Li D., Ito Y., Liu X., Zhang J., Wu C. (2004). An ocular drug delivery system containing zinc diethyldithiocarbamate and HPbetaCD inclusion complex--corneal permeability, anti-cataract effects, and mechanism studies. J. Pharm. Pharmacol..

